# Scalable One-Step Assembly of an Inexpensive Photoelectrode for Water Oxidation by Deposition of a Ti- and Ni-Containing Molecular Precursor on Nanostructured WO_3_

**DOI:** 10.1002/chem.201302641

**Published:** 2013-08-14

**Authors:** Yi-Hsuan Lai, Timothy C King, Dominic S Wright, Erwin Reisner

**Affiliations:** [a] Christian Doppler Laboratory for Sustainable SynGas Chemistry, Department of Chemistry, University of CambridgeLensfield Road, Cambridge CB2 1EW (UK) E-mail: reisner@ch.cam.ac.uk

**Keywords:** photochemistry, photooxidation, photosynthesis, water oxidation, water splitting

Water oxidation is a key challenge to a future energy technology that utilizes solar energy to split water or to reduce carbon dioxide.[1] Photocatalyzing this demanding four-electron, four-proton process efficiently remains an unresolved task and a topic of much current interest.[2] A photo-O_2_ evolution system requires the efficient combination of light harvesting, multi-charge separation and water-oxidation catalysis; all of which are coupled superbly in natural photosynthesis.[3]

Although considerable progress was made recently in the assembly of improved water-oxidizing photoanodes,[4] they typically rely on the use of expensive materials and/or non-scalable fabrication procedures. However, scalable low-cost strategies are required to allow wide adoption of such systems. A promising and emerging approach to form surface-immobilized water-oxidation electrocatalysts is the deposition of molecular precursors on a conducting or semiconducting substrate. For example, Co-containing compounds, as well as a Mn-based molecule, were recently used for the formation of a CoO_*x*_ and a Mn-based water-oxidation electrocatalyst in a pH-neutral environment.[5] We were interested to investigate if this approach could be extended to fabricate an O_2_-evolving photoelectrode by depositing a molecular heterobimetallic precursor on a semiconductor for the simultaneous formation of an electrocatalyst and a stabilizing layer for the substrate.

Herein, we report the assembly of a water-oxidizing photoanode in a straightforward and simple procedure by spin-coating of [Ti_2_(OEt)_9_(NiCl)]_2_ (TiNi) on a nanostructured WO_3_ (nanoWO_3_) electrode (Figure 1). TiNi serves as a molecular single-source precursor for both NiO_*x*_, which acts as the electrocatalyst, and TiO_2_, which stabilizes the WO_3_ semiconductor. The resulting nanoWO_3_|TiNi electrode contains solely Earth-abundant materials and photo-oxidizes water to O_2_, with WO_3_ acting as the solar-light harvesting semiconductor.[6] The one-step co-deposition of a protecting layer, such as TiO_2_ and water oxidation catalyst, such as NiO_*x*_, is an attractive approach to improve photoelectrochemical (PEC) water oxidation.

**Figure 1:**
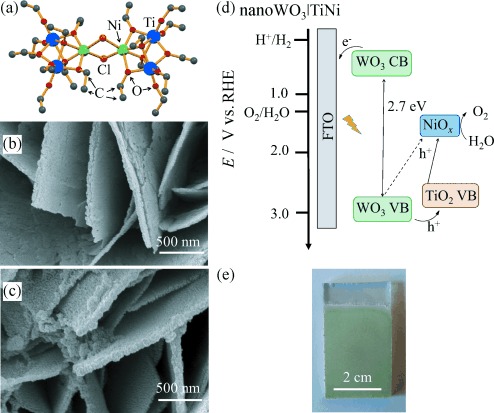
a) Molecular structure of [Ti_2_(OEt)_9_(NiCl)]_2_ (TiNi) based on crystallographic coordinates (hydrogen atoms and disordered ethoxy groups are omitted for clarity).[7] Ni (green), Ti (blue), Cl (orange), O (red), C (grey). SEM images of b) unmodified nanoWO_3_ and c) nanoWO_3_|TiNi. d) Schematic energy diagram for solar-light-driven water oxidation with nanoWO_3_|TiNi. e) Photograph image of nanoWO_3_|TiNi.

The precursor TiNi contains a dimeric [Ni(μ-Cl)_2_Ni]^2+^ bridged core with two attached [Ti_2_(OEt)_9_]^−^ moieties (Figure 1 a) and was readily obtained through a solvothermal reaction of Ti(OEt)_4_ with NiCl_2_.[7] We first assessed the hydrolytic decomposition of TiNi into TiO_2_ and the electroactivity of NiO_*x*_. A water-oxidizing electrode (FTO|TiNi) was assembled by drop-casting TiNi in toluene (10 μL of a 5 mm solution) on a fluoride-doped tin oxide (FTO)-coated glass substrate with an exposed geometrical surface area of 0.5 cm^2^. Hydrolysis and polycondensation of TiNi gave a mixture of amorphous TiO_2_ (Figure S1 in the Supporting Information)[8] and NiO_*x*_, which was confirmed by energy-dispersive X-ray (EDX) analysis (Ti to Ni ratio of ca. 2 to 1; Table S1 in the Supporting Information) and electrochemical investigations. NiO_*x*_ is a known electrocatalyst for water oxidation in borate solution,[9] and NiO_*x*_ on FTO|TiNi electro-oxidizes water to O_2_ with approximately 90 % Faradaic efficiency in potassium borate solution (0.1 m, Bi) at pH 9.2 with a potential of 2.0 V versus the reversible-hydrogen electrode (RHE, Figure 2).

**Figure 2:**
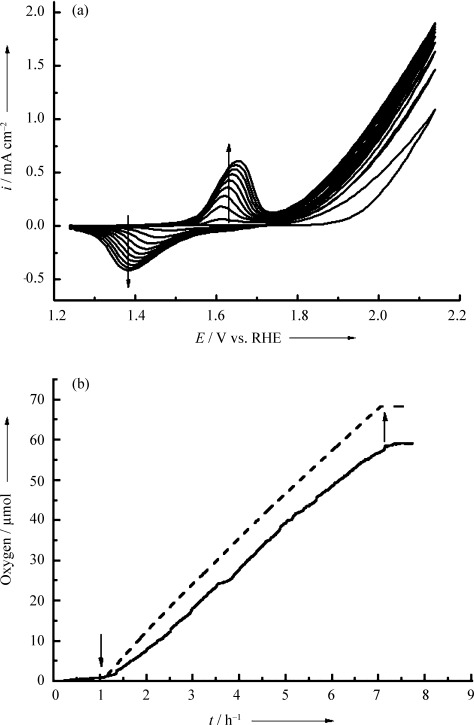
a) Consecutive cyclic voltammograms with FTO|TiNi in an aqueous Bi solution (0.1 m, pH 9.2) at RT and a scan rate of 50 mV s^−1^ showing the increase in the Ni^II/III^ oxidation wave9a, b at approximately *E*_p_=1.62 V versus RHE and the wave for electrocatalytic water oxidation at an onset potential of approximately *E*_cat_=1.73 V versus RHE. A platinum counter and a Ag/AgCl/KCl(sat) reference electrode were employed. b) Amount of O_2_ evolved during controlled potential electrolysis with FTO|TiNi under the same conditions at an applied potential of 2.0 V versus RHE between one and seven hours. The amount of O_2_ was quantified by an O_2_ fluorescence probe (solid trace) and the dashed trace shows the theoretical amount of O_2_ calculated based on 100 % Faradaic efficiency.

WO_3_ is an inexpensive, easily prepared and robust n-type semiconductor with a suitable band structure to absorb visible light (ca. 2.7 eV) and to photo-oxidize water (valence-band potential at ca. 3 V vs. RHE; Figure 1 d).[6, 10] Although the valence band of WO_3_ is not negative enough to achieve hydrogen evolution, it can be coupled with a photocathode to accomplish bias-free overall water splitting.[11] Drawbacks of bare WO_3_ are its chemical dissolution at pH>4,[12] as well as sluggish catalysis and poor selectivity.[13] The slow release of O_2_ also allows competing side reactions, such as the generation of H_2_O_2_, to occur, which causes photodegradation of WO_3_.[13a, 14] Covering metal oxides with an electrocatalyst and/or protective layer based on transitions-metal oxides is a successful strategy to improve their photoactivity and stability,[4b, 5e, 14, 15] and we explore covering WO_3_ with TiNi to improve its performance.

WO_3_ can be prepared by several methods, such as atomic-layer deposition (ALD),[5e] electrodeposition,[14] anodization of tungsten foil,[16] sol–gel synthesis[17] and hydrothermal synthesis.[18] We prepared nanoWO_3_ by the latter method, because it is suitable to prepare vertically aligned nanostructured WO_3_ readily and at a low cost (see the Supporting Information for SEM images and powder XRD patterns; Figures 1 b and S2 in the Supporting Information).[11a, 18a] The sheet-like structure enhances the exposed surface area and decreases the hole diffusion length in nanoWO_3_. NanoWO_3_|TiNi electrodes were prepared by spin coating a toluene solution of TiNi on nanoWO_3_. After four cycles (*N*=4), a quantitative surface coverage of the nanoWO_3_ sheets with TiNi was obtained (see the Experimental Section, Figures 1 c and S3 in the Supporting Information). There was no obvious change in surface morphology of nanoWO_3_ after multiple deposition cycles with TiNi, except that the nanoWO_3_ was decorated with Ti- and Ni-containing nanoparticles, forming a rough and uniform nanoWO_3_|TiNi surface. EDX analyses confirmed a 2:1 to 3:1 stoichiometry of titanium and nickel on the WO_3_ surface (Table S1 and Figure S4 in the Supporting Information).

WO_3_ electrodes are typically only studied under acidic conditions due to the poor photostability of the semiconductor in a basic environment.[5e, 14, 16–18] Previously, a nanostructured WO_3_ electrode prepared by ALD was modified with a Mn-based catalyst and displayed activity for photocatalytic water oxidation between pH 4 and 7.[5e] A planar WO_3_ electrode modified with CoO_*x*_ was also reported to show high photostability in an aqueous phosphate solution at pH 7.[14]

We decided to study the enhanced performance of the nanoWO_3_|TiNi electrodes in an alkaline environment to demonstrate that coating with TiNi can stabilize WO_3_ under such demanding conditions. Our rationale for improved photoactivity and stability of nanoWO_3_|TiNi was that TiO_2_ would serve as a charge-separation layer for transferring holes from the photoexcited WO_3_, thereby decreasing the rate of charge recombination.[19] In addition, TiO_2_ is a known alkaline-resistant material and can at least partly protect WO_3_ from direct contact with the basic solution. NiO_*x*_ is an active water-oxidation catalyst in basic borate solution (see above),[9] and should act as the electro-catalyst driven by photogenerated holes from the valence band of WO_3_. We note that at least some NiO_*x*_ is likely to be in close contact with WO_3_, and hole transfer is therefore also possible to NiO_*x*_ directly from WO_3_ (Figure 1 d).

Photocurrents were measured in a three-electrode configuration with a platinum foil counterelectrode and a Ag/AgCl/KCl(sat) reference electrode at RT, using standardized solar-light irradiation (AM 1.5G, 100 mW cm^−2^). In pH 9.2 Bi solution (0.1 m) at an applied potential of 0.94 and 1.23 V versus RHE, bare nanoWO_3_ showed an initial photocurrent of 131 and 430 μA cm^−2^ with 28±1 and 10±2 % of the photocurrent remaining after 1 h, respectively (Figure 3). An increasing number of TiNi deposition cycles (*N*) resulted in enhanced photostability with 73±3 and 58±3 % of the photocurrent remaining after 1 h continuous irradiation with *N*=4 at 0.94 and 1.23 V versus RHE, respectively (Figures 3 and S5–S6 in the Supporting Information). A half-life time of more than 4 h was found at an applied potential of 0.94 V versus RHE in pH 9.2 Bi solution in the case of nanoWO_3_|TiNi (Figure S7 in the Supporting Information), whereas nanoWO_3_ had lost 50 % of its photoactivity after 35 min. Control experiments involving spin coating Ni(NO_3_)_2_ in 2-methoxyethanol (nanoWO_3_|Ni(NO_3_)_2_, 30 μL, 10 mm) and titanium isopropoxide, [Ti(O*i*Pr)_4_] in toluene (30 μL, 20 mm) on nanoWO_3_ (nanoWO_3_|[Ti(O*i*Pr)_4_]) resulted in stabilities between those for bare nanoWO_3_ and nanoWO_3_|TiNi electrodes. This observation demonstrates that NiO_*x*_ acts as an electrocatalyst and TiO_2_ provides a protective layer (Figures 3 a and S5 in the Supporting Information). The same general trend was also observed in a pH 8.2 Bi electrolyte solution (Figure S8 in the Supporting Information).

**Figure 3:**
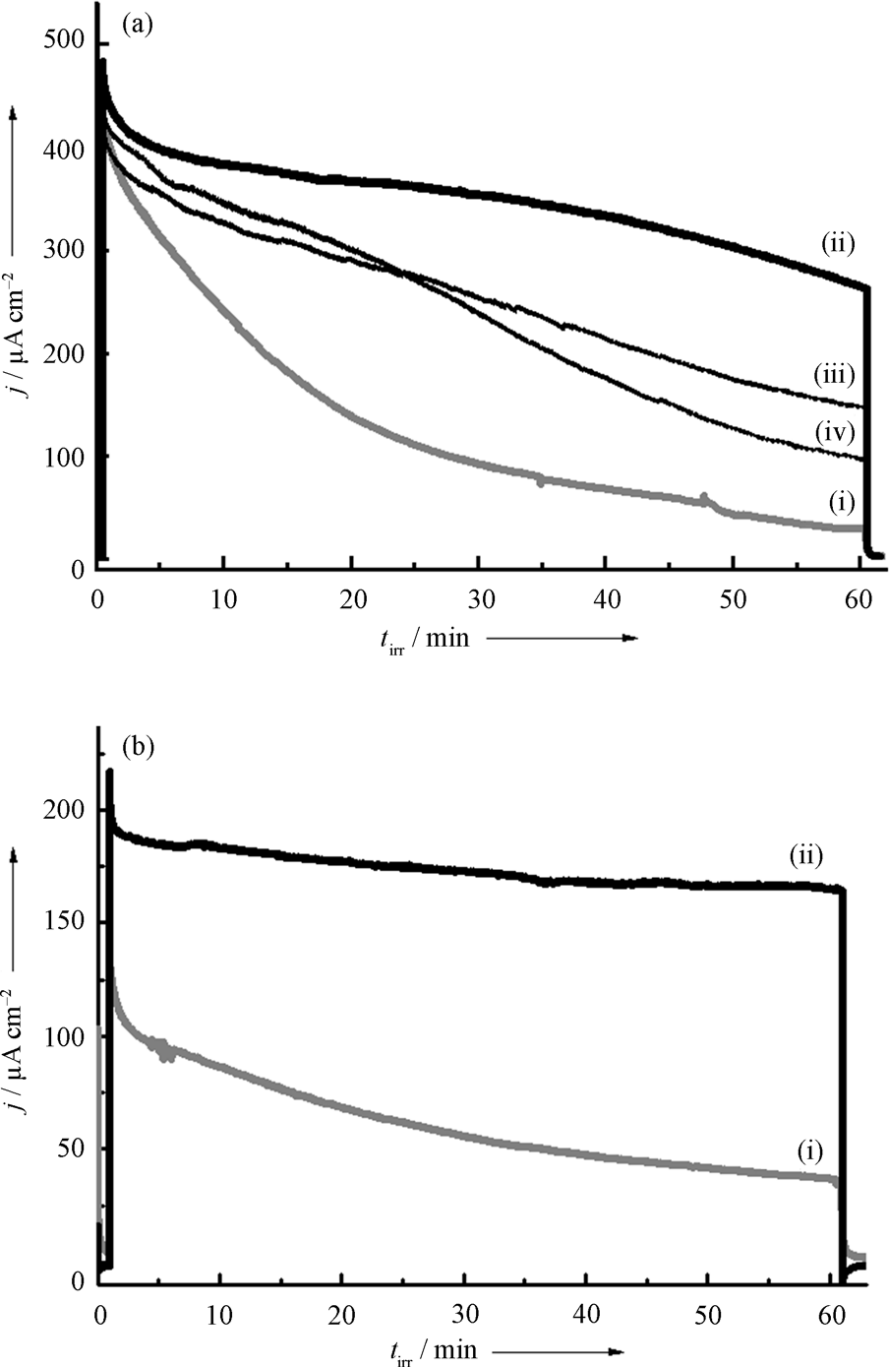
Chronoamperometric measurements a) at 1.23 and b) 0.94 V versus RHE in a pH 9.2 Bi buffer. Photocurrent profiles of i) nanoWO_3_, ii) nanoWO_3_|TiNi, iii) nanoWO_3_|Ni(NO_3_)_2_ and iv) nanoWO_3_|[Ti(O*i*Pr)_4_] under standardized solar-light irradiation (AM 1.5 G, 100 mW cm^−2^) are shown.

The amount of O_2_ and H_2_ (from the platinum counterelectrode in an air-tight three-electrode two-compartment cell) liberated into the headspace of the anodic and cathodic compartments during irradiation was measured by using a fluorescence O_2_ sensor and gas chromatography, respectively (see the Supporting Information, Table S2 and Figure S9). An average charge of 1.17±0.16 and 0.49±0.05 C cm^−2^ h^−1^ was passed through the nanoWO_3_|TiNi and nanoWO_3_ electrodes, respectively, after 1 h irradiation at pH 9.2 and 1.23 V versus RHE. The corresponding Faradaic efficiencies for O_2_ evolution were 74±3 % (with 2.2±0.3 μmol O_2_ cm^−2^ h^−1^) for nanoWO_3_|TiNi and 56±2 % (with 0.71±0.06 μmol O_2_ cm^−2^ h^−1^) for bare nanoWO_3_. Comparable Faradaic yields of 78±1 % (4.70±0.65 μmol H_2_ cm^−2^ h^−1^) and 77±2 % (1.94±0.28 μmol H_2_ cm^−2^ h^−1^) were obtained for H_2_ evolution on the platinum counterelectrode by using nanoWO_3_|TiNi and nanoWO_3_, respectively. The H_2_/O_2_ ratio is therefore close to 2:1 for the nanoWO_3_|TiNi system, whereas it is larger than the ideal 2:1 ratio by using bare nanoWO_3_. The decreased charge generated by bare nanoWO_3_ presumably stems from its poor stability in basic Bi solution, which also results in non-stoichiometric O_2_ evolution and suggests that a considerable portion of the photogenerated holes are used for side reactions. This limitation is largely offset by nanoWO_3_|TiNi. Based on the amount of O_2_ evolution and TiNi on the surface of nanoWO_3_, the turnover frequency of NiO_*x*_ is approximately 8×10^−4^ s^−1^ at 1.23 V versus RHE (see the Supporting Information).

Studying the photocurrents of the nanoWO_3_ electrodes at different potentials at pH 9.2 provided a more comprehensive understanding of the TiNi modification, in particular of the efficiency for photo-water oxidation at a low over-potential. The bare nanoWO_3_ electrode showed an onset photocurrent at 0.74 V versus RHE and the photocurrent increases by applying a more positive potential (Figure 4 a, trace i). The photocurrent saturates at approximately 500 μA cm^−2^ at 1.34 V versus RHE.

**Figure 4:**
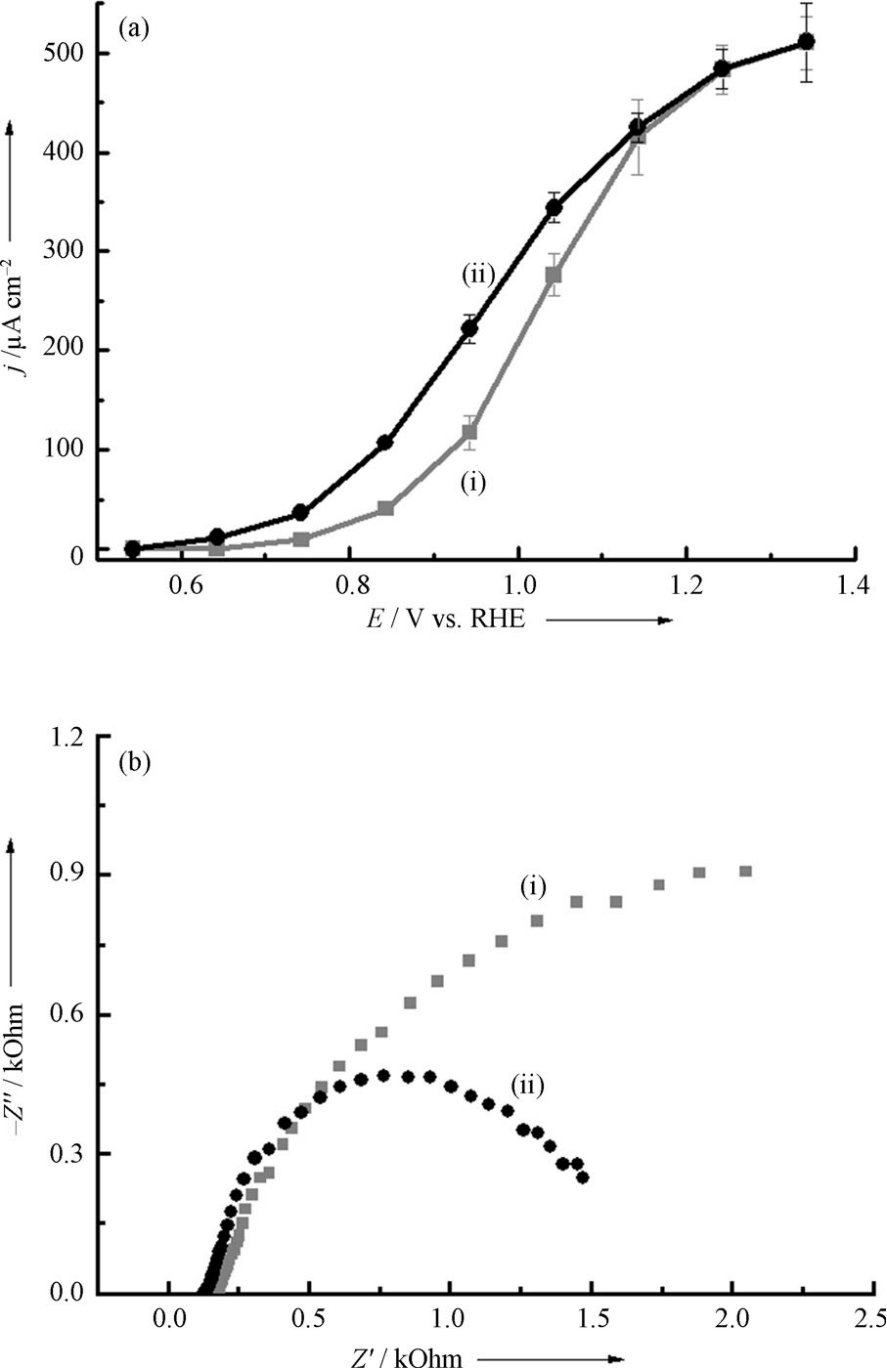
a) Photocurrent responses at various potentials and b) the Nyquist plots at an applied potential of 0.84 V versus RHE of i) an unmodified nanoWO_3_ electrode and ii) a nanoWO_3_|TiNi electrode under standardized solar-light irradiation (AM 1.5 G, 100 mW cm^−2^) in an aqueous Bi solution (0.1 m, pH 9.2).

Modification of nanoWO_3_ with TiNi resulted in an approximately 100 mV cathodic shift of the onset potential (Figure 4 a, trace ii). The effect of the TiNi deposition is particularly evident from enhanced anodic photocurrents in the low bias region (<1.15 V vs. RHE). For example, 107±2 μA cm^−2^ was obtained with nanoWO_3_|TiNi at 0.84 V vs. RHE, whereas only 41±5 μA cm^−2^ was observed with bare nanoWO_3_ at the same potential. A decreased charge-transfer resistance with nanoWO_3_|TiNi was also confirmed by electrochemical impedance spectroscopy at this potential (Figure 4 b). A significant contribution from UV band-gap excitation of TiO_2_ to the total photocurrent density with nanoWO_3_|TiNi can be ruled out, because a comparable photocurrent enhancement was observed both in the presence and absence of a 420 nm UV cut-off filter with nanoWO_3_|TiNi and unmodified WO_3_. A significant enhancement in photocurrent was also observed when modifying nanoWO_3_ with [Ti(O*i*Pr)_4_] in the presence of 420 nm cut-of filter at 0.84 V versus RHE, suggesting that TiO_2_ improves charge separation on the photoanode.

In summary, a nanoWO_3_|TiNi electrode, which is readily prepared from inexpensive materials by using a simple single-source approach, was reported. This technique allows coating of a semiconductor substrate with an electrocatalyst and a stabilizing layer by using a homogeneous, heterobimetallic precursor in one-step. The nanoWO_3_|TiNi electrode showed enhanced water-oxidation catalysis, suffers from fewer limitations from charge recombination than unmodified nanoWO_3_ and allows for the employment of WO_3_ under basic conditions. Our approach can be widely applied to other nanostructured semiconductors and redox reactions. Work is currently in progress in exploring other single-source precursors on different semiconductors to produce new photoactive nanocomposite materials.

## Experimental Section

**Preparation of FTO|TiNi electrode**: The water-oxidation electrode was prepared by drop-casting fresh solutions of TiNi (10 μL of 5 mm in toluene) on fluoride-doped tin oxide (FTO; Pilkington; TEC Glass 7; sheet resistance 7 Ohm sq^−1^) coated glass (exposed surface area of 0.5 cm^2^ controlled by 1350F polyester tape, 3 M). The FTO|TiNi electrode was left at least for 30 min in air at RT, whereupon the electrode was rinsed with water.

**Preparation of nanoWO_3_|TiNi electrode**: The water-oxidation photoelectrode was prepared by spin coating a fresh solution of TiNi (30 μL of 5 mm in toluene) on nanoWO_3_ (exposed area: 0.5 cm^2^) at 2 000 rpm for 10 s. This procedure was repeated *N* times. The nanoWO_3_|TiNi electrode was dried for at least 30 min in air at RT and then washed with water prior to use. For comparison, nanoWO_3_|Ni(NO_3_)_2_ and nanoWO_3_|[Ti(O*i*Pr)_4_] were prepared by spin coating nickel(II) nitrate hexahydrate (Ni(NO_3_)_2_**⋅**6 H_2_O, 30 μL of 10 mm in 2-methoxyethanol; BDH Chemical) and a titanium isopropoxide solution ([Ti(O*i*Pr)_4_], 30 μL of 20 mm in toluene; 97 %; Sigma–Aldrich) on nanoWO_3_ according to the same procedure.

**Electrochemical and PEC measurements**: An Ivium CompactStat potentiostat by using a conventional three-electrode system was employed. FTO|TiNi, nanoWO_3,_ nanoWO_3_|TiNi, nanoWO_3_|Ni(NO_3_)_2_ and nanoWO_3_|[Ti(O*i*Pr)_4_] were used as the working electrodes (all with exposed area of 0.5 cm^2^). A Ag/AgCl/KCl(sat) electrode was used as the reference electrode, and a platinum foil as the counterelectrode. All electrode systems were measured at RT in an aqueous potassium borate solution (Bi, pH 9.2 or pH 8.2). The potentials were converted to the reversible hydrogen electrode (RHE) by using the following Equation:


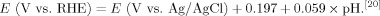


A solar-light simulator (Newport Oriel, 150 W) was used as a light source. The light intensity was adjusted to 100 mW cm^−2^ (1 sun), and an air mass 1.5 global filter and an IR water filter were used.

**Detection and quantification of O_2_ and H_2_**: Electrochemical and PEC water oxidation were carried out by using an electrochemical cell with two compartments separated by a film of Nafion. Headspace O_2_ and H_2_ were quantified by using an Ocean Optics fluorescence oxygen probe (FOXY-R) and/or a gas chromatograph. A potential of 2.0 V versus RHE (no compensation for iR drop) was applied for electrocatalytic water oxidation, whereas a potential of 1.23 V versus RHE for PEC water oxidation. Note that the total amount of O_2_ evolved was determined as the sum of O_2_ measured in the headspace by using the ideal-gas law plus dissolved O_2_ in the solution calculated by Henry’s Law. Please see the Supporting Information for more detailed descriptions.

## Supplementary material

As a service to our authors and readers, this journal provides supporting information supplied by the authors. Such materials are peer reviewed and may be re-organized for online delivery, but are not copy-edited or typeset. Technical support issues arising from supporting information (other than missing files) should be addressed to the authors.
