# Supplementary material for: Scalable One-Step Assembly of an Inexpensive Photoelectrode for Water Oxidation by Deposition of a Ti- and Ni-Containing Molecular Precursor on Nanostructured WO3
Source: Chemistry. 2013 Aug 14;19(39):12943–7. doi: 10.1002/chem.201302641 (PMC3814423; doi:10.1002/chem.201302641)
Supplement: Supplementary file 1 [file chem0019-12943-SD1.pdf]

CHEMISTRY
A EUROPEAN JOURNAL

Supporting Information

© Copyright Wiley-VCH Verlag GmbH & Co. KGaA, 69451 Weinheim, 2013

Scalable One-Step Assembly of an Inexpensive Photoelectrode for Water Oxidation by Deposition of a Ti- and Ni-Containing Molecular Precursor on Nanostructured WO_3

Yi-Hsuan Lai, Timothy C. King, Dominic S. Wright, and Erwin Reisner*[a]

chem_201302641_sm_miscellaneous_information.pdf

Contents

Experimental Section page S2

References page S4

Supporting Tables S1–S2 page S5

Supporting Figures S1–S9 page S6

Experimental Section.

Preparation of $[Ti_2(OEt)_9(NiCl)]_2$ (TiNi). $[Ti_2(OEt)_9(NiCl)]_2$ (TiNi) was synthesized as reported previously by heating titanium ethoxide (7.00 mL, 33.4 mmol; 99.99%; Sigma-Aldrich), nickel(II) chloride (271 mg, 2.09 mmol; >97%; Sigma-Aldrich) in anhydrous ethanol (7.00 mL, 120 mmol) in a Teflon-lined autoclave at 150 ^{o}C for 24 h.[1] Dark yellow crystals were collected upon evaporation of ethanol and crystallization in dry toluene at –14 ^{o}C.

Preparation of a *nano*WO_3 electrode. *Nano*WO_3 was synthesized following a published procedure.[2] To prepare WO_3 seed layer modified FTO, a precursor solution containing H_2WO_4 (0.625 g, 99%; Sigma-Aldrich), polyvinyl alcohol (0.5 g, 98-99%, medium molecular weight; Alfa Aesar) in H_2O_2 (10 mL, 30%; Sigma-Aldrich) was prepared and spin-coated on FTO-coated glass followed by annealing in air for 2 h at 500 ^{o}C with a Carbolite® furnace. For growth of the *nano*WO_3 sheets on FTO, a second precursor solution was prepared by adding H_2WO_4 (3 mL of 0.25 M) in H_2O_2 to an aqueous HCl solution (3 mL, 1 M) containing oxalic acid (0.2g, 99%; Sigma-Aldrich). Then, acetonitrile (10 mL, HPLC grade; Fisher Chemicals) was added to this acidic solution. Subsequently, this precursor solution was put in a 23 mL Teflon-lined stainless steel autoclave (model 4749, Parr). The WO_3 seed layer-modified FTO substrate was vertically immersed into the second precursor solution in the autoclave,

whereupon the autoclave was sealed and heated at 180 °C for 2.5 h. After growth of the WO_3 nanosheets, the resultant electrodes were rinsed with ethanol, followed by annealing in air for 1 h at 500 °C. SEM images confirm that the FTO substrate is fully covered with a WO_3 nanosheet film (Figures 1b and S2a; sheet thickness is approximately 50 nm) and powder X-ray diffraction (XRD) analysis confirms a monoclinic WO_3 phase (Figure S2b).

Physical characterization. The surface morphologies of FTO|TiNi, *nano*WO_3 and *nano*WO_3|TiNi electrodes were characterized using a Phillips XL30-SFGE scanning electron microscope (SEM) and the elemental analyses of the films were studied using an x-sight light element energy dispersive X-ray (EDX) spectrometer (Phillips XL30-SFGE). Atomic percentages and standard deviations where calculated by the built-in EDAX software. The XRD analyses for the electrodes were carried out using an X'Pert PRO X-ray diffractometer (PANalytical B.V., The Netherlands). UV-vis absorption spectra were recorded on a Varian Cary 50 UV-vis spectrophotometer. The amount of Ti and Ni on WO_3 was quantified by analysing $[TiCl_6]^{2-}$ after dissolution for two h in concentrated aqueous HCl by UV-vis spectrophotometry at λ = 308 nm and assuming a Ti to Ni ratio on *nano*WO_3 of roughly 2 to 1. The amount of Ni on *nano*WO_3 is approximately 0.78 ± 0.07 μmol cm^{-2}.

Oxygen measurement. Oxygen was analysed in the headspace of the anodic compartment of the PEC cell by using an Ocean Optics fluorescence oxygen probe (FOXY-R). The probe was inserted through a tightly sealed septum and continuous O_2 readings (O_2 partial pressure) at 1 s intervals were made throughout the experiment. For electrocatalytic O_2 production, we employed an FTO|TiNi working, a platinum counter and a Ag/AgCl/KCl(sat) reference electrode. A potential of 2.0 V *vs.* RHE (no compensation for iR drop) was applied between one and seven h of the experiment. The first and eighth h were used as control with no applied

potential. For photoelectrochemical O_2 production by *nano*WO_3 and *nano*WO_3|TiNi, a *nano*WO_3 or *nano*WO_3|TiNi was used as working, whereas platinum and Ag/AgCl/KCl(sat) electrodes were used as counter and reference electrodes, respectively. The cell was operated at an applied voltage of 1.23 V *vs.* RHE in the dark during the first 60 min (control experiment), followed by 60 min under standardized light illumination (100 mW cm^{-2}) at an applied voltage of 1.23 V, and another 60 min in the dark at an applied voltage of 1.23 V (control experiment). For both electrochemical and photoelectrochemical water oxidation, the total amount of O_2 evolved was determined as the sum of O_2 measured in the headspace using the ideal gas law plus dissolved O_2 in the solution calculated by Henry's Law.

Hydrogen measurement. The amount of H_2 generated in the headspace of the cathodic compartment was detected and quantified with an Agilent 7890A Series GC equipped with a 5 Å molecular sieve column (N_2 carrier gas at a flow rate of approximately 3 mL min^{-1}). The GC oven kept the columns at 45 ºC, and a thermal conductivity detector was used. The electrochemical cell was purged with 2% CH_4 in N_2 for at least 20 min prior to PEC experiments; methane acts as an internal standard for H_2 quantification by gas chromatography (GC). 40 μL aliquots of the headspace gas were removed from the airtight electrochemical cell for GC analysis after PEC experiments.

References

[1] S. Eslava, M. McPartlin, R. I. Thomson, J. M. Rawson, D. S. Wright, *Inorg. Chem.* **2010**, *49*, 11532-11540.

[2] a) C.-Y. Lin, Y.-H. Lai, D. Mersch, E. Reisner, *Chem. Sci.* **2012**, *3*, 3482-3487; b) J. Su, X. Feng, J. D. Sloppy, L. Guo, C. A. Grimes, *Nano Lett.* **2011**, *11*, 203-208.

Table S1. EDX results of FTO|TiNi, *nano*WO_3|TiNi (N = 1) and *nano*WO_3|TiNi (N = 4).

	Element content (atomic %)	
	Ti	Ni
FTO|TiNi	1.03 ± 0.1	0.63 ± 0.2
nanoWO_3|TiNi (N = 1)	0.90 ± 1.5	0.43 ± 0.1
nanoWO_3|TiNi (N = 4)	2.98 ± 1.7	0.91 ± 0.2

Table S2. Photoelectrochemical O_2 produced by *nano*WO_3|TiNi and *nano*WO_3 in a pH 9.2 Bi buffer at 1.23 V *vs.* RHE under solar light illumination (100 mW cm^{-2}, AM 1.5G). The amount of H_2 produced by platinum counter electrode was also determined.

Electrode	Charge / C	O_2 / μmol	O_2 FE / %	H_2 / μmol	H_2 FE/ %
nanoWO_3|TiNi					
1 (1.5 cm^2)	1.60	3.10	76.4	6.40	78.5
2 (2.0 cm^2)	2.15	4.21	73.2	8.74	77.2
3 (2.0 cm^2)	2.70	5.13	71.8	10.9	78.0
			74 ± 3[a]		78 ± 1[a]
nanoWO_3					
1 (1.5 cm^2)	0.76	1.10	55.8	3.05	77.3
2 (2.0 cm^2)	1.06	1.51	54.9	4.33	78.8
3 (2.0 cm^2)	0.88	1.30	57.0	3.27	74.1
			56 ± 2[a]		77 ± 2[a]

[a] Average Faradic efficiency (FE) ± standard deviation (σ)

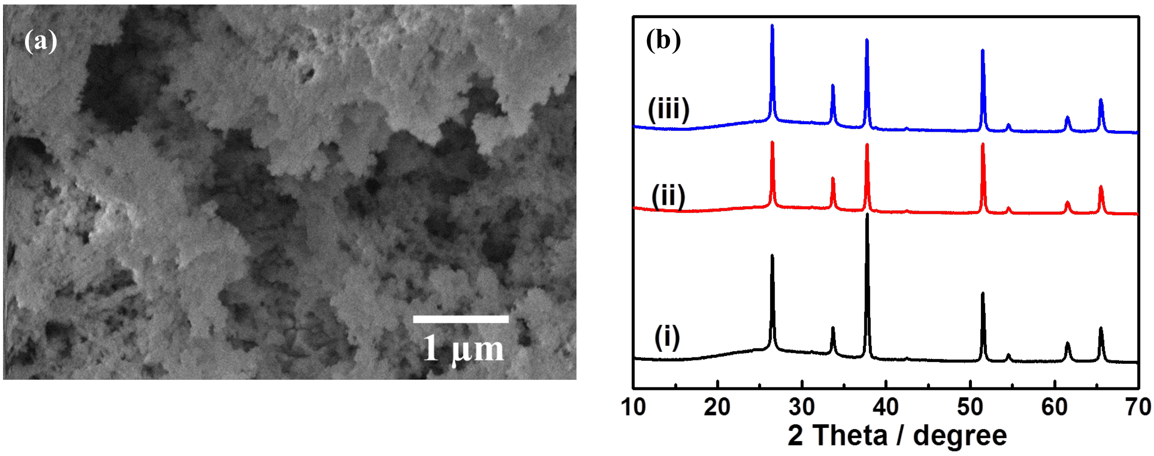

Figure S1. (a) SEM image of FTO|TiNi showing that the electrode is composed of agglomerated particles. (b) XRD patterns of (i) unmodified FTO, (ii) as-prepared FTO|TiNi and (iii) after electrochemical treatment of FTO|TiNi. No additional peaks were observed in as-prepared FTO|TiNi and electrochemical treated FTO|TiNi, indicating the TiO_2 and NiO_x deposits are amorphous.

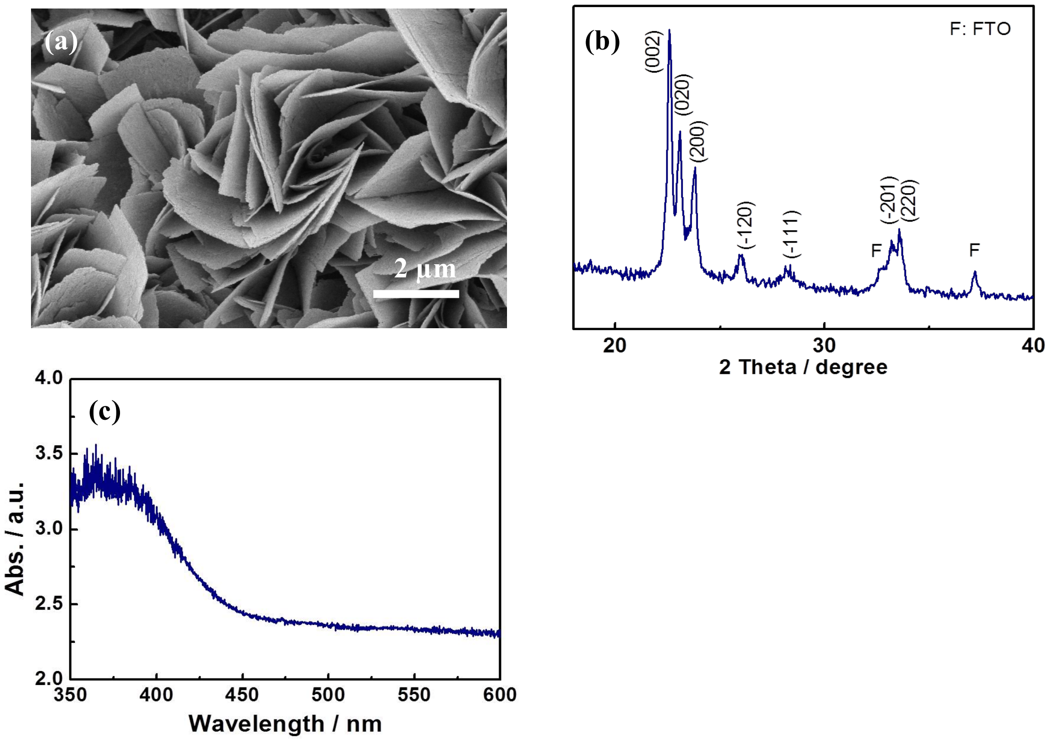

Figure S2. (a) Surface morphology (SEM image), (b) XRD analysis, and (c) UV-vis absorption spectrum of unmodified *nano*WO_3.

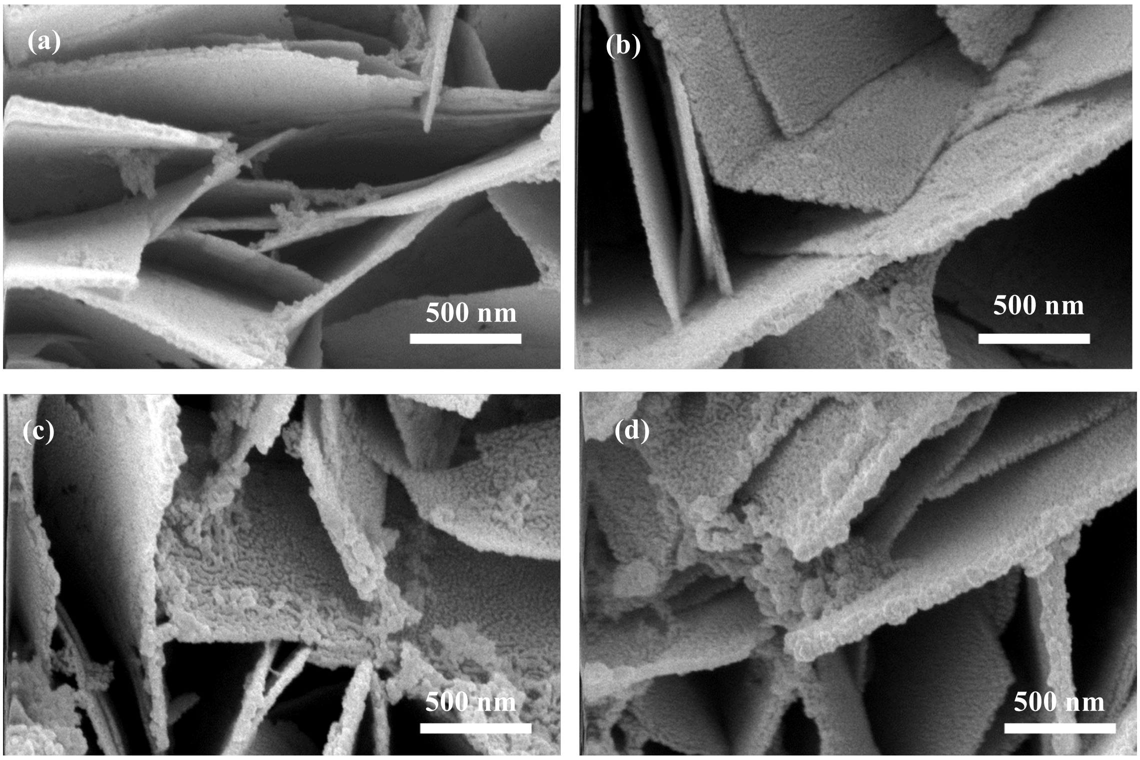

Figure S3. SEM images of a (a) *nano*WO_3|TiNi (N = 1), (b) *nano*WO_3|TiNi (N = 2), (c) *nano*WO_3|TiNi (N = 3) and (d) *nano*WO_3|TiNi (N = 4).

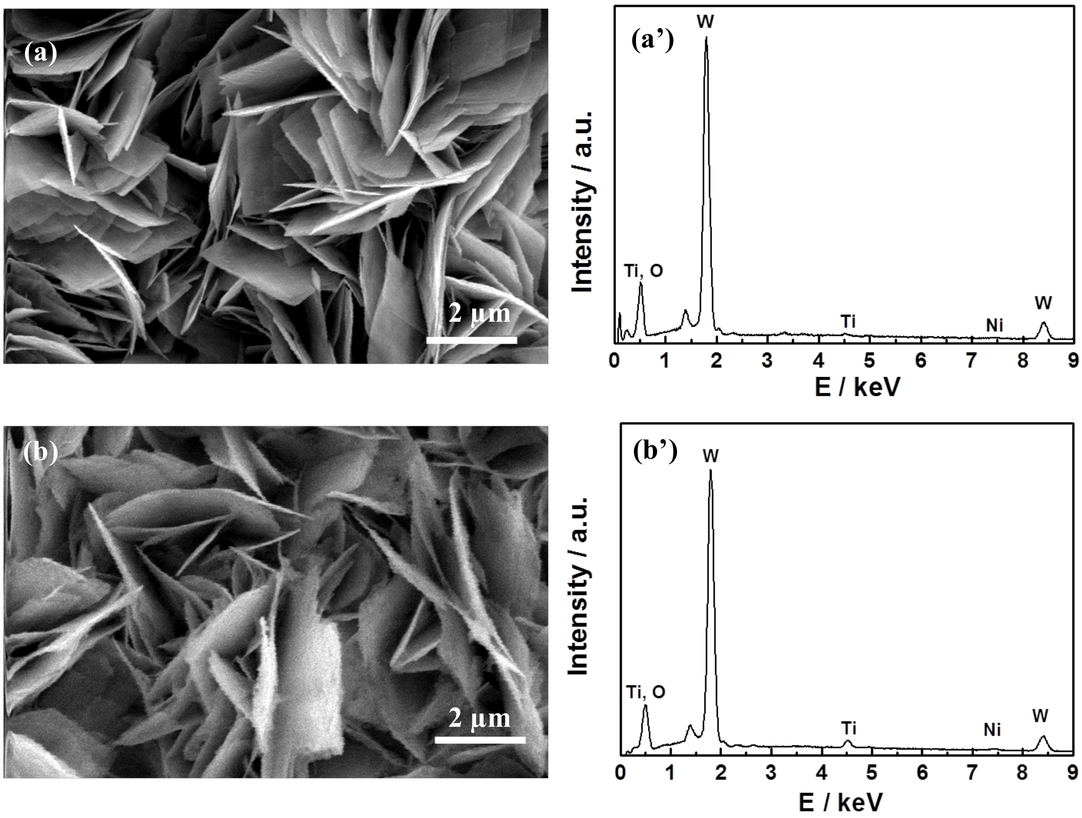

Figure S4. (a) Top view SEM image of a *nano*WO_3|TiNi (N = 1) electrode and (a’) its corresponding EDX spetrum. (b) Top view SEM image of a *nano*WO_3|TiNi (N = 4) electrode and (b’) its corresponding EDX spetrum.

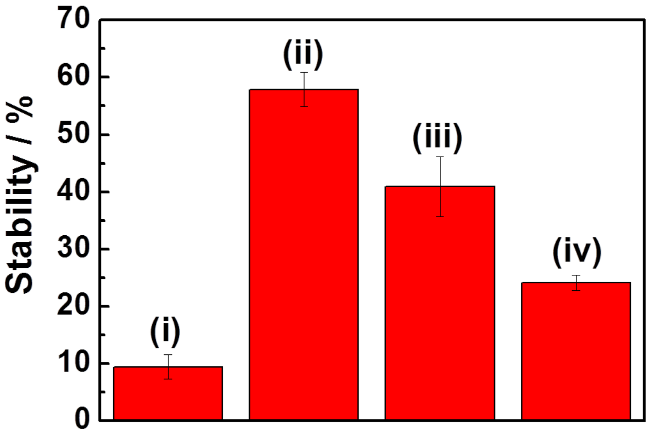

Figure S5. Summary of the relative photoactivity at 1.23 V *vs.* RHE in a pH 9.2 Bi buffer (0.1 M) under AM 1.5G light illumination (100 mW cm^{-2}) after 60 min operation for a (i) *nano*WO_3, (ii) *nano*WO_3|TiNi (N = 4), (iii) *nano*WO_3|$Ni(NO_3)_2$ (N = 4) and (iv) *nano*WO_3|[$Ti(O^iPr)_4$] (N = 4) electrode.

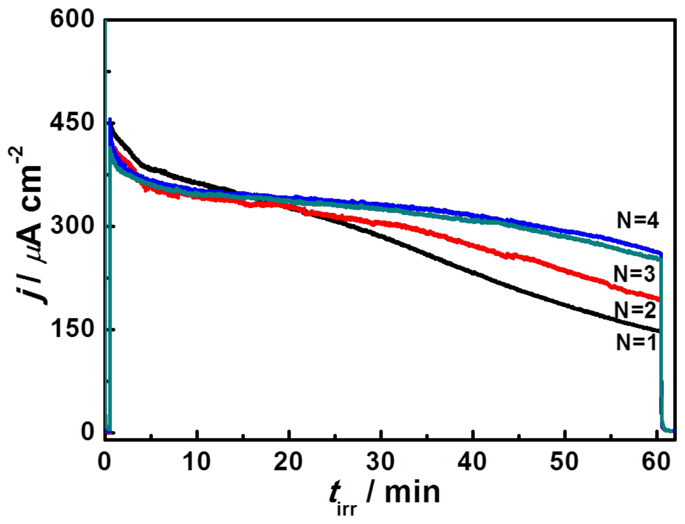

Figure S6. Photocurrent profiles of a *nano*WO_3|TiNi (N = 1), *nano*WO_3|TiNi (N = 2), *nano*WO_3|TiNi (N = 3) and *nano*WO_3|TiNi (N = 4) electrode at 1.23 V *vs.* RHE in an aqueous Bi buffer solution (0.1 M, pH 9.2) under standardized solar light irradiation (AM 1.5 G, 100 mW cm^{-2}).

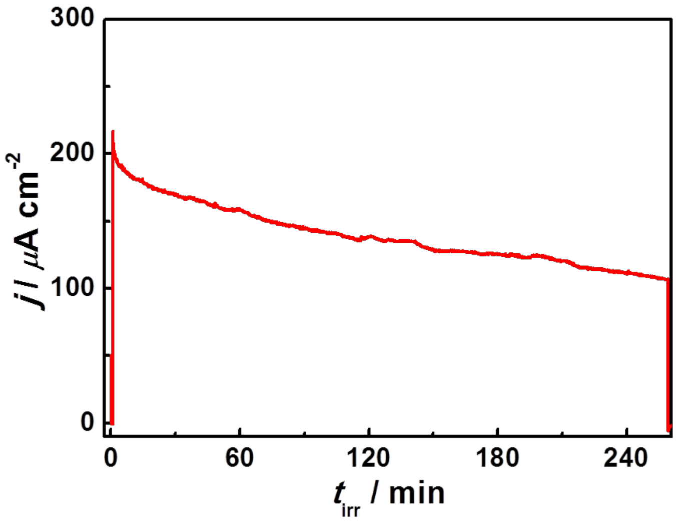

Figure S7. The photocurrent transient of a *nano*WO_3|TiNi (N = 4) electrode at 0.94 V *vs.* RHE in an aqueous Bi buffer (0.1 M, pH 9.2) and AM 1.5G (100 mW cm^{-2}) irradiation.

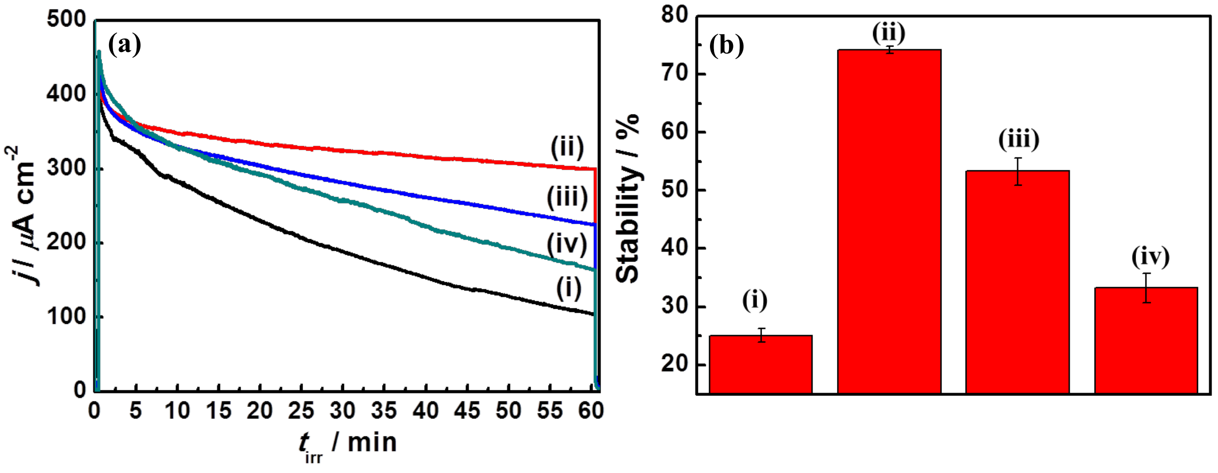

Figure S8. (a) Photocurrent profiles of a (i) *nano*WO_3, (ii) *nano*WO_3|TiNi (N = 1), (iii) *nano*WO_3|$Ni(NO_3)_2$ (N = 1) and (iv) *nano*WO_3|[$Ti(O^iPr)_4$] (N = 1) electrode measured at 1.23 V *vs.* RHE in a pH 8.2 Bi buffer (0.1M) under AM 1.5G light illumination (100 mW cm^{-2}). (b) Summary of the relative activity after 60 min operation in pH 8.2 Bi buffer of (a).

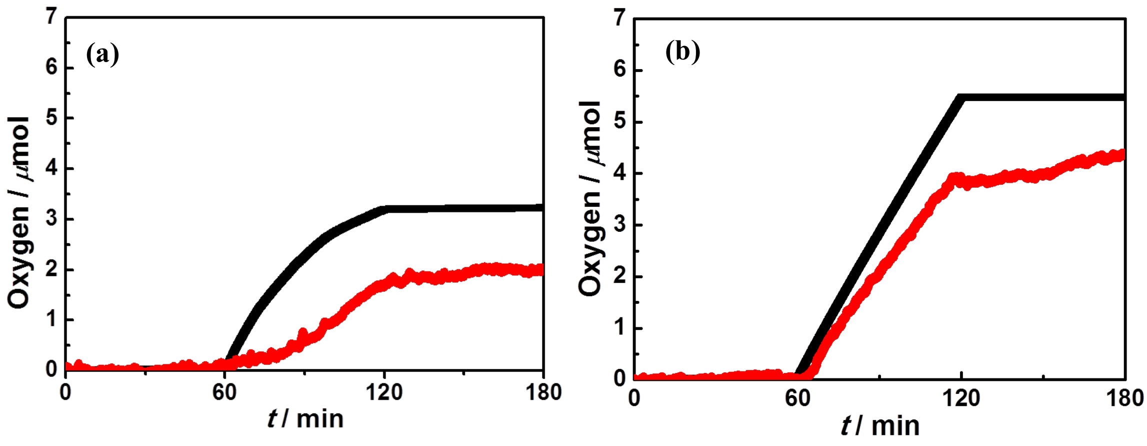

Figure S9. Photo-O_2 generated by a (a) *nano*WO_3 and (b) *nano*WO_3|TiNi (N = 4) with an applied bias of 1.23 V *vs.* RHE with standardized solar light irradiation (AM 1.5G, 100 mW cm^{-2}) between 60 and 120 min. O_2 was detected in the headspace by a fluorescence based sensor and the amount of headspace O_2 was corrected for dissolved O_2 in solution using Henry's Law (red trace). The black trace shows the theoretical amount of O_2 produced during irradiation based on the photocurrent with 100% Faradaic yield.

- End of Supporting Information -
